# Supplementary material for: Impact of Partitioning in Short-Term Food Contact Applications Focused on Polymers in Support of Migration Modelling and Exposure Risk Assessment
Source: Molecules. 2021 Dec 26;27(1):121. doi: 10.3390/molecules27010121 (PMC8746823; doi:10.3390/molecules27010121)
Supplement: Supplementary file 1 [file molecules-27-00121-s001.zip › molecules-1498729-supplementary.pdf]

molecules

Supplementary data

Impact of partitioning in short-term food contact applications focused on polymers in support of migration modelling and exposure risk assessment

Rainer Brandsch [1], Mark Pemberton [2], Dieter Schuster [1], Frank Welle [3,*]

[1] SAFE+ Algorithmics GmbH, Untere Läng 8c, 82205 Gilching, Germany; rainer.brandsch@saferithm.com, dieter.schuster@saferithm.com
[2] Systox Limited, Sutton Grange, Parvey Lane, Sutton, Cheshire, SK11 0HX, United Kingdom; markpemberton@systox.com
[3] Fraunhofer Institute for Process Engineering and Packaging (IVV), Giggenhauser Straße 35, 85354 Freising, Germany; welle@ivv.fraunhofer.de
* Correspondence: welle@ivv.fraunhofer.de

1. Generic Diffusion Coefficient Approach

Figure S1 to Figure S3 shows the predicted migration in dependence of diffusion coefficients D_P ranging from 10^{-10} cm²/s to 10^{-15} cm²/s and partition coefficients ranges $K_{P,F}$ = 0.1 to 10000 for several thicknesses of the packaging material (100 µm, 200 µm and 1000 µm) with an initial concentration $c_{P,0}$ = 100 mg/kg in the material. The surface volume ratio was set V = 1000 ml and A = 600 cm².

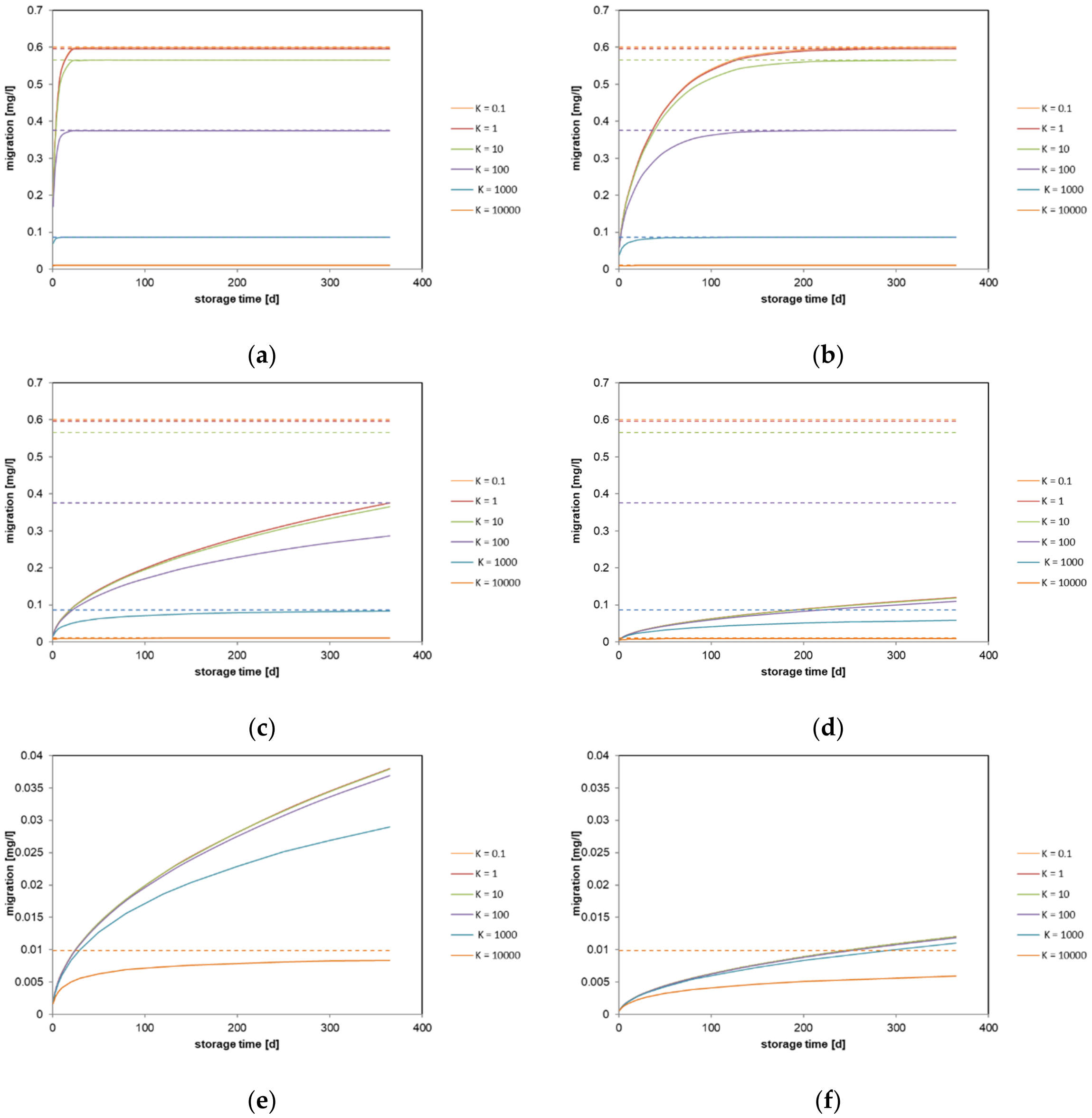

Figure S1. Calculated migration into food considering different partition coefficients: (a) diffusion coefficient DP = 1 × 10^{-10} cm^2/s, (b) DP = 1 × 10^{-11} cm^2/s, (c) DP = 1 × 10^{-12} cm^2/s, (d) DP = 1 × 10^{-13} cm^2/s, (e) DP = 1 × 10^{-14} cm^2/s and (f) DP = 1 × 10^{-15} cm^2/s. Calculated for a layer thickness of d = 100 µm, cP,0 = 100 mg/kg, V = 1000 mL, A = 600 cm^2. Note: (e) and (f) different scale on y-axis.

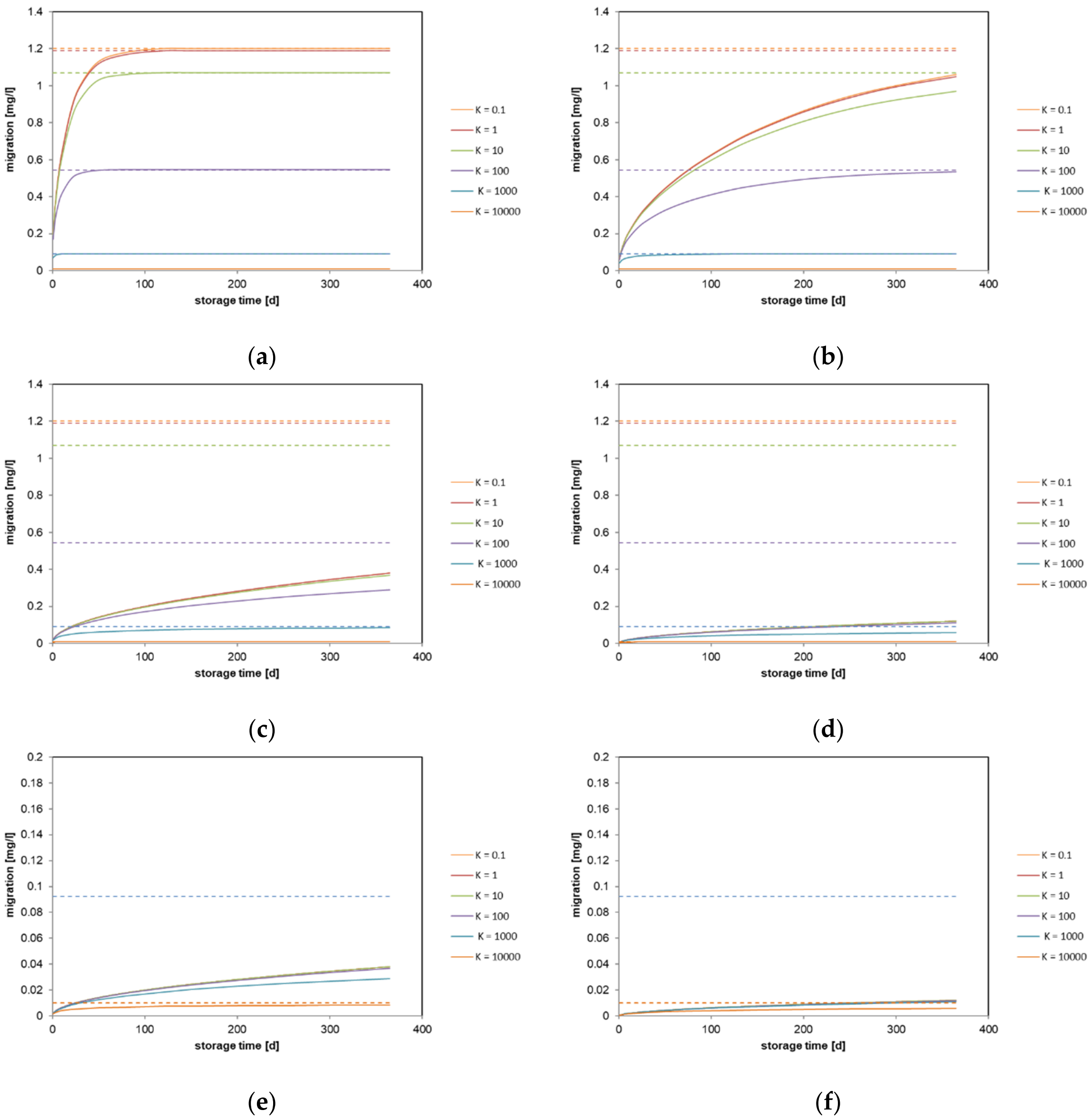

Figure S2. Calculated migration into food considering different partition coefficients: (a) diffusion coefficient DP = 1 × 10^{-10} cm^2/s, (b) DP = 1 × 10^{-11} cm^2/s, (c) DP = 1 × 10^{-12} cm^2/s, (d) DP = 1 × 10^{-13} cm^2/s, (e) DP = 1 × 10^{-14} cm^2/s and (f) DP = 1 × 10^{-15} cm^2/s. Calculated for a layer thickness of d = 200 µm, cP,0 = 100 mg/kg, V = 1000 mL, A = 600 cm^2. Note: (e) and (f) different scale on y-axis.

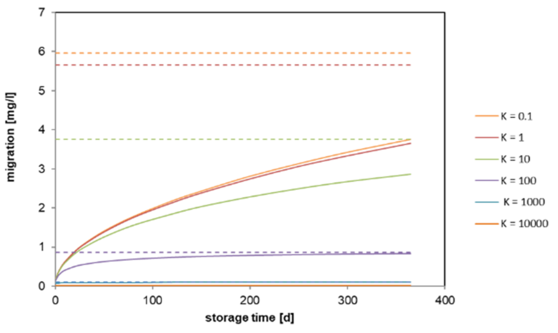

(**a**)

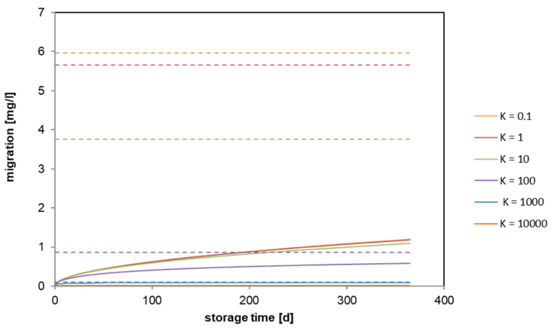

(**b**)

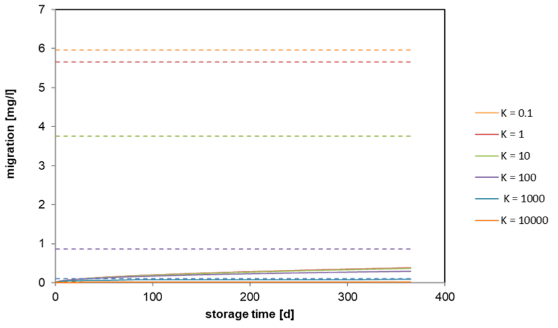

(**c**)

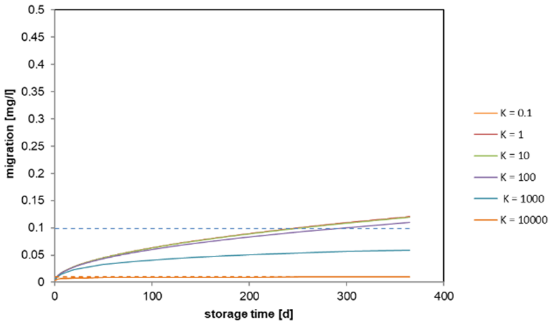

(**d**)

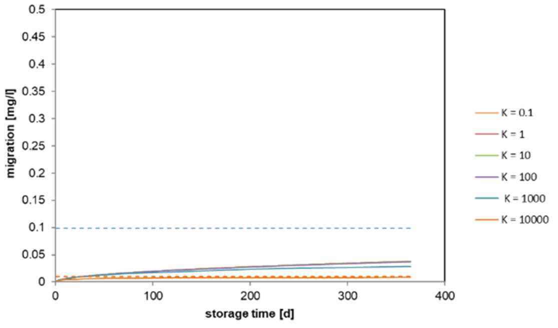

(**e**)

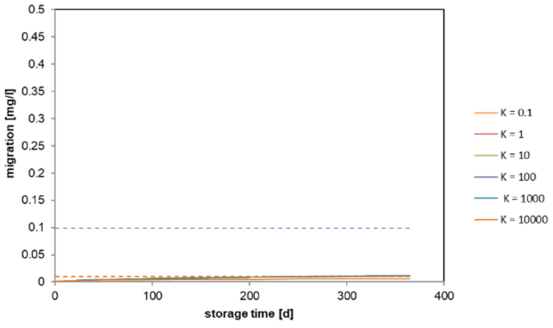

(**f**)

Figure S3. Calculated migration into food considering different partition coefficients: (a) diffusion coefficient DP = 1 × 10^{-10} cm^2/s, (b) DP = 1 × 10^{-11} cm^2/s, (c) DP = 1 × 10^{-12} cm^2/s, (d) DP = 1 × 10^{-13} cm^2/s, (e) DP = 1 × 10^{-14} cm^2/s and (f) DP = 1 × 10^{-15} cm^2/s. Calculated for a layer thickness of d = 1000 μm, cP,0 = 100 mg/kg, V = 1000 mL, A = 600 cm^2. Note: (e) and (f) different scale on y-axis.
